# Case Report: A Pregnant Woman Diagnosed as ALK-Rearrangement Lung Large Cell Neuroendocrine Cancer With Brain Metastasis

**DOI:** 10.3389/fonc.2022.823813

**Published:** 2022-02-25

**Authors:** Zaixiang Fu, Ganggui Zhu, Liquan Wang, Shen Hu, Lu Cheng, Fuyi Liu

**Affiliations:** ^1^Department of Neurosurgery, Second Affiliated Hospital, School of Medicine, Zhejiang University, Hangzhou, China; ^2^Department of Obstetrics, Second Affiliated Hospital, School of Medicine, Zhejiang University, Hangzhou, China; ^3^Department of Pathology, Second Affiliated Hospital, School of Medicine, Zhejiang University, Hangzhou, China

**Keywords:** ALK rearrangement, ALK inhibitors, alectinib, large cell neuroendocrine carcinoma, pregnancy, brain tumor, NGS

## Abstract

Concomitant malignant tumors and pregnancy present many difficult questions to both clinicians and patients. Due to no specific guidelines, each aspect of clinical management requires special considerations. This current report presents a rare case of a 38-year-old pregnant woman at gestational age 33 weeks with complaints of weakness of her right limbs for 2 weeks. After successive cesarean section and craniotomy, a diagnosis of lung large cell neuroendocrine carcinoma (LCNEC) metastatic to the brain was eventually made. Next generation sequencing (NGS) showed ALK-EML4 gene fusion. Immediately afterwards she was started on the targeted therapy with the ALK inhibitor alectinib. Ten months later, all known lesions exhibited a rapid regression, and no new brain metastases were found. Consequently, the therapeutic effect was considered as a partial response. Then, we review the previous literature using PubMed on maternal malignant brain tumors diagnosed during pregnancy, or lung LCNEC associated with ALK fusion, or ALK inhibitors treatment among the pregnant women, eventually, and discuss the concerns of dealing with these patients.

## Introduction

It is very rare for a woman diagnosed as lung cancer with brain metastasis during pregnancy. Because of the special physiological condition of the mother and fetus and the low prevalence of such tumors, no standard treatment guidelines are published. Pulmonary large cell neuroendocrine carcinoma (LCNEC) is a rare subtype of lung cancer with aggressive behavior and poor prognosis, and the incidence appeared to be approximately 3% in a series of surgically resected cases (1). Anaplastic lymphoma kinase (ALK) fusion genes can be detected in approximately 5%–6% of all non-small cell lung cancer (NSCLC) patients, especially lung adenocarcinoma (2), thus, there are fewer patients with ALK rearrangement in LCNEC. To our knowledge, no studies have reported ALK rearrangement lung LCNEC with brain metastasis during pregnancy. Herein, we describe such a case and review the related literature.

## Case Presentation

**Figure 1:**
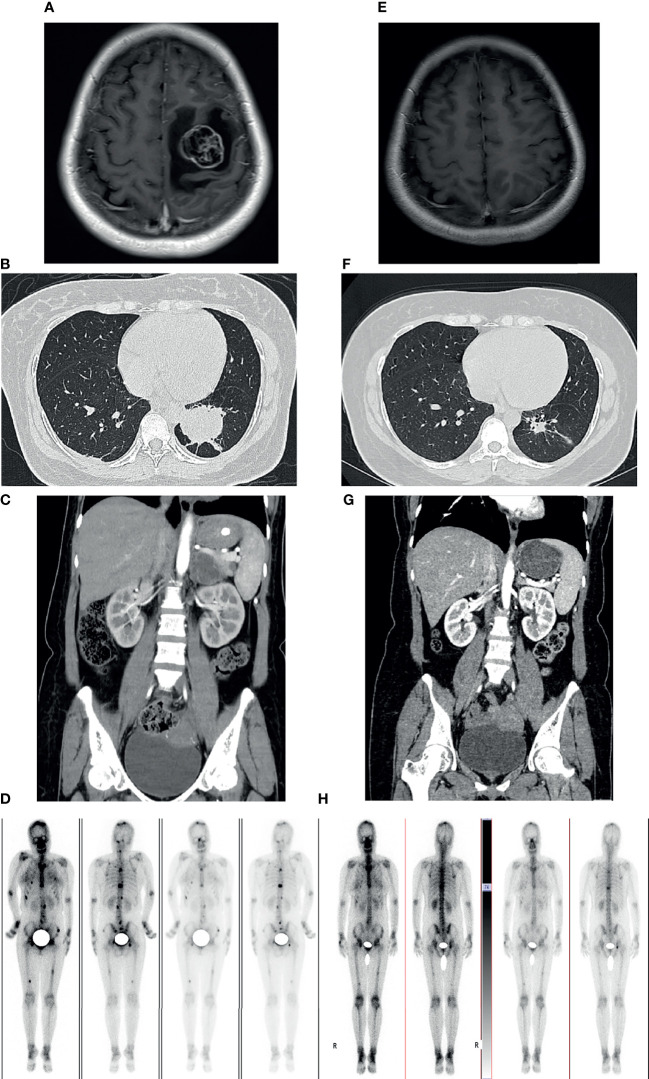
Imaging examinations before craniotomy and alectinib treatment **(A–D)**. 10-months follow-up radiological evaluation **(E–H)**. **(A)** Contrast-enhanced cranial MRI showed the lesion in the left frontal lobe. **(B)** CT scan of the chest showing the mass in left lower lobe of the lung. **(C)** CT enhancement scan showing the mass in the left adrenal. **(D)** 99mTc-MDP bone scintigraphy demonstrating multiple lesions of increased activity in spine, pelvis, skull, ribs, skull and femurs. **(E–H)** Radiological evaluation demonstrating a dramatical shrinkage of all known lesions after 10 months.

**Figure 2:**
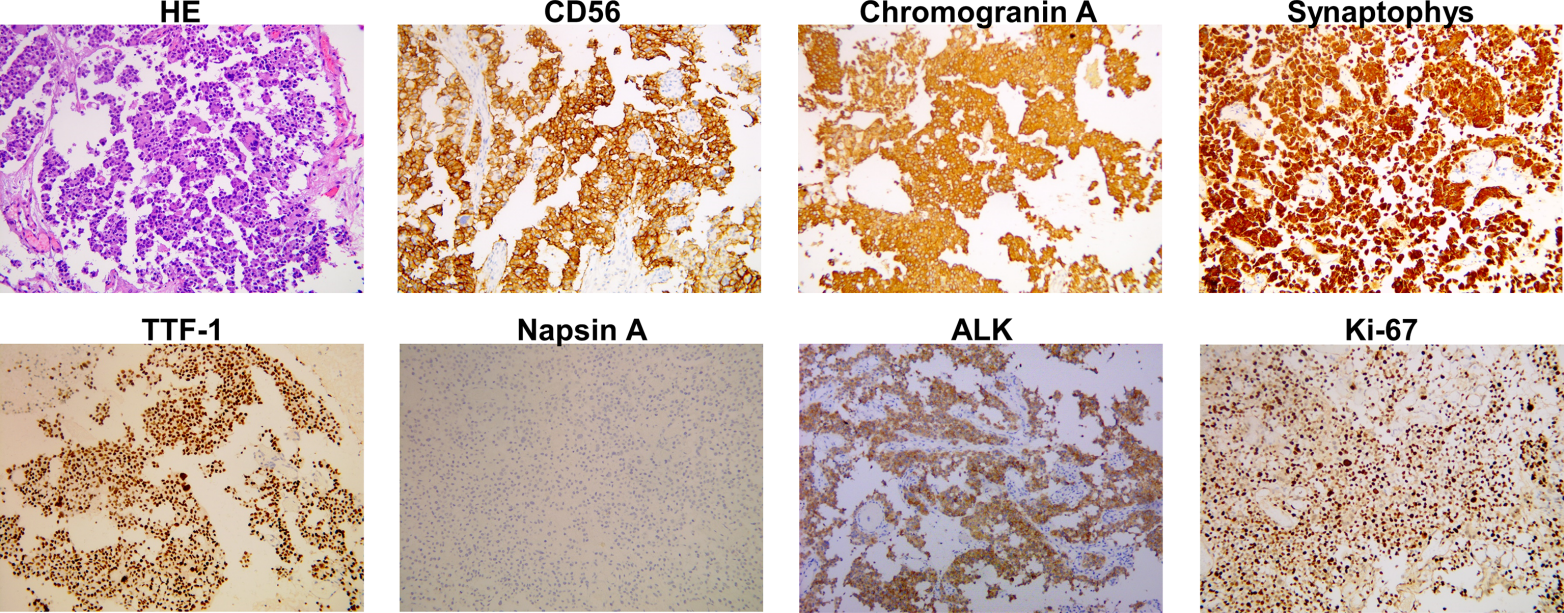
Histopathological findings of the tumor in left frontal lobe. Haematoxylin and eosin (HE) staining is shown. Immunostaining indicated positivity for CD56, chromogranin A, synaptophys, thyroid transcription factor-1 (TTF-1), ALK, and negative for napsin A. The Ki67 staining index is 70%.

A previously healthy 38-year-old woman (gravida 1 para 0, frozen embryo transfer) who was 33 weeks pregnant was referred to the Obstetric Department of our hospital with a history of progressive numbness and weakness of her right limbs for 2 weeks. The day before admission, magnetic resonance imaging (MRI) was performed at the local hospital to consider neoplastic lesions in the left frontal lobe. Physical examination showed that the muscle strength of the right upper limb was grade 0 and the right lower limb was grade 3. On the 1st day of admission, the consultation of obstetrics, pediatrics and neurosurgery concluded that the patient was most likely to suffer from intracranial malignant tumor (metastasis or primary tumor). Since the fetus was in good condition at 33 weeks of gestation, it was proposed to promote fetal lung maturation at first, terminate the pregnancy by cesarean section at 34 weeks, and then perform craniotomy to remove the tumor as soon as possible. Considering the influence of contrast agent on the fetus, enhanced MRI examination was planned to be performed after termination of pregnancy. But the patient had rapid clinical deterioration. So an emergency cesarean section was performed under intravertebral anesthesia on the 2nd day. A healthy baby boy was delivered safely. The fetus was preterm and no neonatal malformations were found. After delivery, contrast-enhanced MRI of brain showed a 30.3*28.8mm brain cystic solid mass in the left frontal lobe; accompanied by severe peritumoral edema; the solid and edge of lesion showed enhancement following administration of a contrast agent (**Figure 1**). On the 4th day, emergency craniotomy was performed because of the increased intracranial pressure, and then the patient was transferred to the Department of Neurosurgery. Macroscopically, the tumor is reddish in color and soft in texture with rich blood supply. The tumor was removed completely and sent for pathological examination. Immunohistochemistry (ICH) showed that TTF-1, Syn, CgA, CD56, AE1/AE3, and CK7 were positive, while NapsinA, P40, PAX-8, and GATA-3 were negative (**Figure 2**). The Ki67 proliferation index was 70%. Subsequently, a chest CT scan revealed a 45*36*50mm tumor in the lower lobe of the left lung, and an abdominal CT showed a 39*41mm metastasis in the left adrenal gland (**Figure 1**). ECT demonstrated multiple bone metastases throughout the body (**Figure 1**). The pathological diagnosis was poorly differentiated neuroendocrine tumor, consistent with LCNEC, and the clinical stage was determined to be IV. In order to determine the optimal therapeutic strategy, next generation sequencing (NGS) of tumor samples and patient blood showed ALK-EML4 gene fusion with a mutation frequency of 29.21%. Anti-ALK ICH was performed to confirm ALK protein expression (**Figure 2**). Therefore, the patient was treated with rehabilitation and alectinib, a novel highly selective inhibitor of ALK translocation. After 10 months of treatment with alectinib, the symptoms gradually improved, and radiological evaluation showed a dramatical shrinkage of all known lesions (**Figure 1**). At present, the patient is raising her child as normal at home and her performance status (PS) is 1 without any major adverse events.

## Discussion

The main characteristics of this case were: (1) the woman was diagnosed as a malignant intracranial tumor at 33 weeks of gestation and developed progressive neurological deterioration;(2) the tumor was pathologically diagnosed as LCNEC and NGS of ctDNA (circulation tumor DNA) showed EML4-ALK fusion;(3) the patient took alectinib treatment after delivery and the lesions shrunk dramatically.

Firstly, the incidence of most primary brain tumor in pregnancy seems not to be higher except for choriocarcinomas, meningiomas, and pituitary adenomas (3, 4). And there are no extracranial tumors that are likely to metastasize that are uniquely related to the specific pregnancy (5). But certain factors such as immunological tolerance, hormone-mediated growth, and hemodynamic changes may promote neoplasm growth mediating a common pathway to increasing intracranial mass effect (3, 4, 6). Intracranial tumors usually initially present with symptoms and signs of increased intracranial pressure such as headache, dizziness, or vomiting, related focal neurological deficits and seizures, which are often confused with pregnancy itself, hypertensive disorders and thrombosis in pregnancy (7). Many of patients are misdiagnosed and fail to receive timely treatment. Therefore, it is vital for clinicians to pay attention to such patient with prolonged, non-remission and worsening symptoms during pregnancy to avoid ignoring the diagnosis of brain tumors. MRI is the preferred auxiliary examination because of its greater sensitivity, the best soft tissue visualization and lack of ionizing radiation. A gadolinium-containing contrast agent should be treated with caution, and it may be used only if the fetus and mother significantly benefit from it (8). In the acute setting, clinicians should not withhold head CTs. The fetal dose exposure from the maternal head CT is about 0.001-0.01 mGy which is lower than the minimum estimated threshold dose(50 mGy) (8).

In addition, due to the lack of level I or II evidence, most of comes from case reports and experts’ opinions, so the development of treatment plan requires multidisciplinary collaboration between the obstetrician, neurosurgeon, anesthetist and so on. The major challenging questions include medical therapy, the timing of surgical intervention, the timing and type of delivery, and the mode of anesthesia. About medical management, steroids are the most important component of medical treatment, which can not only alleviate vasogenic cerebral edema and but also facilitate fetal lung maturity (9). Although long-term steroid use can contribute to neonatal hypoadrenalism, it is an uncommon complication (10). Mannitol has a risk of affecting fetal circulation, and doses of 0.5–1 g/kg are considered safe (7). Additionally, prophylactic use of antiepileptic drugs is not recommended because of their teratogenicity. On the other hand, what we are most concerned about is the timing of surgery and delivery in pregnancy. In general, it depends on tumor type, gestational age, patient’s clinical status and preferences. We reviewed the relevant literature, summarized the algorithm of N Kitchen (4) and Tewari et al (11), and made the following recommendations for patients of an antenatal malignant symptomatic brain tumor (1). If the patient is in the early first trimester of pregnancy, a therapeutic abortion may be an option, since the risks of surgery, radiation therapy or chemotherapy are too high to the fetus while delayed treatment may be unsafe for the mother (2, 9). In stable patients presenting in the first or early second trimester, with a strong desire to continue the pregnancy, gestational advancement may be permitted to the early second trimester prior to neurosurgery and radiotherapy. Unstable patients require emergency craniotomy and the risk of fetal loss must be clarified (3). The ideal time for surgical intervention seems to be the second trimester because of fetal vulnerability during the first and increased maternal intravascular volume in the third trimester (12). In the late second trimester and third trimester, stable patients should be closely monitored until fetal maturity. Patients presenting with deteriorating neurological function may be treated with radiotherapy as an option to delay surgery. For unstable patients with a risk of brain herniation, a cesarean section should be performed under general anesthesia, followed by immediately surgical decompression and tumor resection (4). Most obstetricians and neonatologists will postpone delivery until 32 or even 34 weeks of gestation, if possible, to ensure maturity and survival of the fetus.

Secondly, an ALK rearrangement generally occurs in lung adenocarcinoma, accounting for 5%–6% of all NSCLC cases, and is associated with younger age (median age of diagnosis of 55), and non-smoking or less smoking (2). Lung LCNEC is a rare subgroup of pulmonary neuroendocrine carcinoma with high malignancy and a dismal prognosis, appears to be more common in male, older, and heavy smokers (1). We searched the PubMed database and reviewed the previous and this case reports about lung LCNEC patients with ALK rearrangement (13–20) (**Table 1**). Of the ten cases, we found that the median age was 45.5 years (range=32-75 years), 60% were women, and 40% were smokers, which did not show a clear tendency to clinical features, perhaps related to too few cases. Interestingly, 9 of these 10 cases were from Asiatic patients (**Table 1**). However, no a definite racial difference for ALK rearrangement has been reported (21). Instead, epidermal growth factor receptor (EGFR)-activating mutations are more common in East Asians (22, 23). Additionally, these oncogenic driver mutations, including EGFR, ALK, VEGF, HER2, c-KIT and so on, have been reported in LCNEC patients in some literature, particularly in Asiatic patients (24–26). But up to now LCNEC has remained poorly characterized due to its rarity. Therefore, further studies on the relationship between the molecular characteristics such as ALK rearrangement and clinical features of LCNEC may be needed. On the other hand, in contrast to pure NSCLC or LCNEC, we found NSCLC with neuroendocrine differentiation is a distinct and controversial entity (27). Unlike neuroendocrine carcinoma, hematoxylin and eosin (HE) findings of these tumors do not show a neuroendocrine phenotype, while immunohistochemical stains can indicate positivity for neuroendocrine markers such as chromogranin A, synaptophys or CD56 (27). However, on the basis of the current evidence, there is no clear relationship between neuroendocrine differentiation in NSCLC and prognostic implications (27, 28). Of note, Caumont et al. (29) reported a case of ALK-rearrangement pulmonary adenocarcinoma treated with ALK inhibitor (ALK-I) crizotinib that produced neuroendocrine transformation associated with acquired resistance to crizontinib, but Sim et al. (30) found the tumor might be responsive to second generation ALK-Is, which was consistent with case report of Mengoli et al. (31).

**Table 1:** List of cases reported to have lung LCNEC with ALK rearrangement.

No	The first author	Year	Age/sex	Nation	Smoking status	ALK detection	Fusion genes	Clinical stage	ALK Inhbitor Therapy	Clinical outcome	PFS (Months)
1	Omachi (13)	2014	43/F	Japan	N	IHC, FISH, RT-PCR	EML4	IV	Crizotinib	Progression	1.4
2	Hoton (15)	2017	69/F	Turkey	N	FISH	NA	IV	Crizotinib	Progression	6(Crizotinib)
									Ceritinib	Progression	9(Ceritinib)
3	Hayashi (14)	2017	75/F	Japan	N	FISH	NA	IVb	Alectinib	PR	6+
4	Zheng (16)	2018	44/M	China	Y	FISH	NA	IVb	N	NA	NA
5	Zheng (16)	2018	47/F	China	N	IHC, NGS	EML4	IVa	Crizotinib	PR	10+
6	Shimizu (17)	2018	73/M	Japan	Y	FISH, RT-PCR	KIF5B	IVb	Crizotinib	Progression	8 (Crizotinib)
									Alectinib	SD	4+ (Alectinib)
7	Wang (20)	2019	41/M	China	Y	IHC, FISH, NGS	PLB1	IVb	Crizotinib	Progression	5 (Crizotinib)
									Anlotinib		1.4(Anlotinib)
									Ceritinib		4.5 (Ceritinib)
8	Tashiro (18)	2020	32/F	Japan	Y	IHC, FISH	NA	IVb	Alectinib	Progression	11
9	Masuda (19)	2021	72/M	Japan	N	IHC, FISH	NA	IV	Alectinib	PR	1+
										Progression	4
10	This case	2021	38/F	China	N	NGS, IHC	EML4	IV	Alectinib	PR	10+

IHC, immunohistochemistry; FISH, fluorescence in situ hybridization; RT-PCR, reverse transcription polymerase chain reaction; NGS, next-generation sequencing; F, female; M, male; Y, yes; N, no; NA, not available; PR, partial response; SD, stable disease, PFS, progression-free survival; LCNEC, large cell neuroendocrine carcinoma; ALK, anaplastic lymphoma kinase; EML4, echinoderm microtubule-associated protein-like 4; KIF5B, the kinesin family 5B gene; PLB1, phospholipase B1.

Currently, the main diagnostic approaches used to detect ALK fusion include IHC, fluorescence *in situ* hybridization (FISH), RT-PCR, NGS. To date, FISH remains the “gold standard” for diagnosis. But these methods have their own limitations, such as IHC is unable to demonstrate ALK status directly (32), FISH has the disadvantages of operator-dependent, signal instability and low sensitivity (32, 33), and RT-PCR requires high quality RNA (32) and cannot detect unknown fusion partners (32, 34). For instance, because of the physiological expression of ALK in nerve cells, Takeuchi (35) considered that ALK rearrangement by ICH sometimes could be false positive in some lung cancers with neuroendocrine differentiation (30), particularly in LCNEC (36). In contrast, NGS has the highest specificity (37), is increasingly cost effective (32), detect multiple genetic alterations, regardless of known or unknown ALK fusions (32, 33, 37), and can be used with solid or liquid biopsies (38, 39). Therefore, we speculate that NGS will gradually change the standard of ALK testing, especially detect mutations of resistance to ALK-Is.

Thirdly, the co-existence of ALK positive lung cancer and pregnancy is a rare condition. To date, there are no data to assess the molecular and genomic characteristics of these patients. In the USA, many ALK-Is such as crizotinib, alectinib, brigatinib, and ceritinib have been approved for the first line of therapy (40). But little information is available on the efficacy, fetal side effects, and gestational complications of these ALK-Is in pregnant patients. A single-institution, retrospective study published by Dagogo-Jack et al. found that of the eight pregnant women with lung cancer between 2009 and 2015, six had an ALK rearrangement and received ALK-Is treatment after delivery (41). Furthermore, we summarized 9 patients from previous and this case reports (42–49) (**Table 2**) and ALK-Is were used after delivery on six patients. These patients produced very positive results, similar to those of non-pregnant patients, thus we speculate ALK-Is treatment may be successfully used after delivery (43, 50). Significantly, of the nine patients, two were treated with crizotinib for a short period of time at the late pregnancy stage (48, 49), while only one received treatment with alectinib during the entire pregnancy (47). No evidence of abnormal fetal development due to ALK-Is during pregnancy was found. Nevertheless, we cannot exclude some unknown and delayed risks to child development, which requires longer follow-up.

**Table 2:** List of pregnant women reported to have lung cancer with ALK rearrangement.

No	The first author	Year	Age	Nation	Timing of diagnosis (weeks)	Delivery (weeks)	Pathology	TKI treatment	Timing of TKI	Clinical outcome	PFS (months)
1	Neves (45)	2014	36	Portugal	27	29	AD	Crizotinib	After delivery	SD	9
2	Sariman (46)	2013	34	Turkey.	After delivery	28	AD	Crizotinib	After delivery	SD	6+
3	Komura (44)	2018	28	Japan	After delivery	37	AD	Alectinib	After delivery	PR	12+
4	Bellido (43)	2019	42	Spain	30	30	AD	Crizotinib	Puerperium	Progression	2
								Alectinib		SD	10+
5	Acosta Rojas (42)	2020	31	Spain	23	32	AD	Crizotinib	After delivery	Progression	60
6	Scarfone (47)	2021	31	Italy	Before pregnancy	35	NA	Alectinib	Before pregnancy	PR	32+
7	Padrao (48)	2018	36	Portugal	22	30	AD	Crizotinib	26 weeks of gestation	Progression	4
								Ceritinib		Died	2
8	Jensen (49)	2019	32	Denmark	20	26	AD	Crizotinib	23 weeks of gestation	SD	3
										Died	4
9	This case	2021	38	China	After delivery	33	LCNEC	Alectinib	Puerperium	PR	10+

AD, Adenocarcinoma; ALK, anaplastic lymphoma kinase; TKI,tyrosine kinase inhibitors; Y, yes; N, no; NA, not available; PR, partial response; SD, stable disease; PFS, progression-free survival.

## Conclusions

It is a real challenge for clinicians to manage malignant intracranial tumors in pregnant patients, attempting to decide on the optimal strategy to minimize the risk to the mother and fetus. Specialized medical teams with abundant experience and multidisciplinary discussions from the perspectives of the patient’s clinical characteristics as well as preferences are paramount to develop individualized and the best approach. Based on previous reports, even though ALK rearrangement is a relatively rare event in patients with lung LCNEC and/or pregnancy, this phenomenon demonstrated that driver mutations tests are also necessary and NGS may become a mainstream approach in the future. ALK-Is seem to be used successfully after delivery according to some previous case reports. There have been no reports about major fetal side effects or pregnancy complications with ALK-Is during pregnancy or the peripartum period, highlighting the necessity for further investigation.

## Data Availability Statement

The original contributions presented in the study are included in the article/supplementary material. Further inquiries can be directed to the corresponding author.

## Ethics Statement

Written informed consent was obtained from the individual(s) for the publication of any potentially identifiable images or data included in this article.

## Author Contributions

ZF drafted the manuscript and performed the literature review. GZ, LW, and SH retrieved the clinical and the image information. LC provided and analyzed the pathological information. FL designed the study and revised the manuscript. All authors contributed to the article and approved the final version of the manuscript.

## Conflict of Interest

The authors declare that the research was conducted in the absence of any commercial or financial relationships that could be construed as a potential conflict of interest.

## Publisher’s Note

All claims expressed in this article are solely those of the authors and do not necessarily represent those of their affiliated organizations, or those of the publisher, the editors and the reviewers. Any product that may be evaluated in this article, or claim that may be made by its manufacturer, is not guaranteed or endorsed by the publisher.
